# Role of the Gut on Glucose Homeostasis: Lesson Learned from Metabolic Surgery

**DOI:** 10.1007/s11883-017-0642-5

**Published:** 2017-02-09

**Authors:** V. Kamvissi-Lorenz, M. Raffaelli, S. Bornstein, G. Mingrone

**Affiliations:** 1 Department of Medicine 3, Universitätsklinikum Carl Gustav Carus an der Technischen Universität Dresden, Dresden, Germany; 2 Diabetes and Nutritional Sciences, King’s College London, Henr. Rahp. R. 3.6, Guy’s Campus, 19 Newcomen Street, London, SE1 1UL UK; 3 Department of Surgery, Catholic University, Rome, Italy; 4 Department of Internal Medicine, Catholic University, Rome, Italy

**Keywords:** Bariatric surgery, Gastric bypass, Biliopancreatic bypass, Sleeve gastrectomy, Diabetes mellitus, Obesity

## Abstract

**Purpose of Review:**

Bariatric surgery was initially intended to reduce weight, and only subsequently was the remission of type two diabetes (T2D) observed as a collateral event. At the moment, the term “metabolic surgery” is used to underline the fact that this type of surgery is performed specifically to treat diabetes and its metabolic complications, such as hyperlipidemia.

**Recent Findings:**

Randomized, controlled studies have recently supported the use of bariatric surgery, and in particular of Roux-en-Y gastric bypass (RYGB) and biliopancreatic diversion (BPD) as an effective treatment for decompensated T2D. The lesson learned from these randomized and many other non-randomized clinical studies is that the stomach and the small intestine play a central role in glucose homeostasis. Bypassing the duodenum and parts of the jejunum exerts a substantial effect on insulin sensitivity and secretion. In fact, with BPD, nutrient transit bypasses duodenum, the entire jejunum and a small portion of the ileum, resulting in reversal of insulin sensitivity back to normal and reduction of insulin secretion, whereas RYGB has little effect on insulin resistance but increases insulin secretion. Hypotheses concerning the mechanism of action of metabolic surgery for diabetes remission vary from theories focusing on jejunal nutrient sensing, to incretin action, to the blunted secretion of putative insulin resistance hormone(s), to changes in the microbiota.

**Summary:**

Whatever the mechanism, metabolic surgery has the undoubted merit of exposing the central role of the small intestine in insulin sensitivity and glucose homeostasis.

## Introduction

In the last few years, a great deal of attention has been focused on the effects of bariatric surgery on diabetes remission and changes in glucose homeostasis. In fact, a foremost achievement of bariatric surgery has been to uncover the role of the small intestine in glucose metabolism.

The term “bariatric” derives from the Greek word “baros”, meaning weight. Bariatric surgery was in fact developed to cure morbidly obese subjects. The idea of a surgical treatment of obesity developed in the early 1950s fortuitously from the observation that patients that underwent gastrointestinal resections for various reasons were likely to lose weight.

An international consensus conference held in Rome in 2007 - the “Diabetes Surgery Summit”—underlined the need to use the adjective “metabolic” instead of “bariatric” in order to highlight the efficacy of bariatric surgery from the metabolic point of view even in the absence of weight reduction [1]. Indeed, the designation of “metabolic surgery” was previously used by Buchwald and Varco [2] for some operations like the portal diversion to improve glycogen storage diseases or the partial ileal bypass for hyperlipidemia.

In view of the weight independent effects of some types of gastrointestinal surgery for obesity, Rubino [3] proposed to use metabolic surgery not only for uncontrolled T2D, but also for patients with the metabolic syndrome, non-alcoholic steatohepatitis (NASH), and increased cardiovascular risk, presuming a neuroendocrine mechanism of action for this surgery.

Here, we seek to briefly summarize recent findings from randomized trials on the impact of bariatric surgery on metabolic outcomes, and devote the remainder this article to presenting a new perspective on the role played by the small intestine in driving the changes in insulin sensitivity and secretion and glycemic control that occur after some types of bariatric surgery. A better understanding of gut function in glucose disposal might help to develop, in the near future, a medical treatment for T2D that mimics the effects of gastrointestinal surgery.

### Review of Recent Randomized Trials

Randomized controlled trials (RCT) have shown that bariatric/metabolic surgery is effective in treating type 2 diabetes mellitus. An extensive review of the literature at this regard is behind our scope; therefore, we have summarized only the results of some relevant RCTs.

The first evidence of the efficacy of bariatric surgery on T2DM is that from Dixon et al.’s [4] RCT showing that T2DM remission was present in 73% of the patients who underwent LAGB and in 13% of those in the conventional therapy group.

Shauer et al. [5] demonstrated that the proportion of patients achieving a glycated hemoglobin (HbA1c) level ≤6.0% 12 months after treatment was 12% in the medical-therapy group versus 42% in the RYGB (*P* = 0.002) and 37% in the SG group (*P* = 0.008). Therefore, at least at 1 year after surgery there was no difference between the two types of operation. However, the same authors reported that at 3 years following surgery [6], the criterion for the primary end point was met by 5% of the patients in the conventional therapy group and in 38% of those in the RYGB group (*P* < 0.001) and 24% of those in the SG group (*P* = 0.01), thus showing a more sustained effect of the RYGB procedure as compared with SG. Importantly, quality of life was significantly better in the two surgical arms than in the medical arm.

Having as primary end point a diabetes remission rate (defined as a fasting glucose level of <100 mg per deciliter [5.6 mmol per liter] and a glycated hemoglobin level of <6.5% in the absence of pharmacologic therapy) at 2 years after intervention, Mingrone et al. [7] demonstrated that 75% of the patients who had undergone RYGB and 95% BPD had diabetes remission (*P* < 0.001 for both comparisons versus medical-therapy group). However, in the long term (5 years follow-up) 50% of the surgical patients (37% in the RYGB and 63% in the BPD group) maintained diabetes remission (*P* = 0.0007 versus medically treated patients) while the other patients had diabetic relapse [8], although the number of anti-diabetic, anti-hypertensive and hypolipidemic drugs were significantly lower while the quality of life was significantly better in the surgical than in the medical arm.

Metabolic surgery is effective in treating T2DM also in patients with a BMI <35 kg/m^2^ as shown by Cohen et al. [9] at 6 years following RYGB with a durable diabetes remission that occurred in 88% of cases and with glycemic improvement in 11%.

In the Crossroad RCT, Cummings et al. [10] compared RYGB with intensive lifestyle and medical intervention (ILMI) in T2DM patients with a BMI <35 kg/m^2^ and found that diabetes remission (HbA1c <6.0% [<42.1 mmol/mol], off all diabetes medicines) at 1 year was achieved in 60.0% of RYGB and in 5.9% of ILMI (*P* = 0.002).

There RCTs show that bariatric/metabolic surgery is indeed a suitable option to treat patients with uncontrolled T2DM and this independently of their baseline BMI.

### Surgical Procedures

**Fig. 1:**
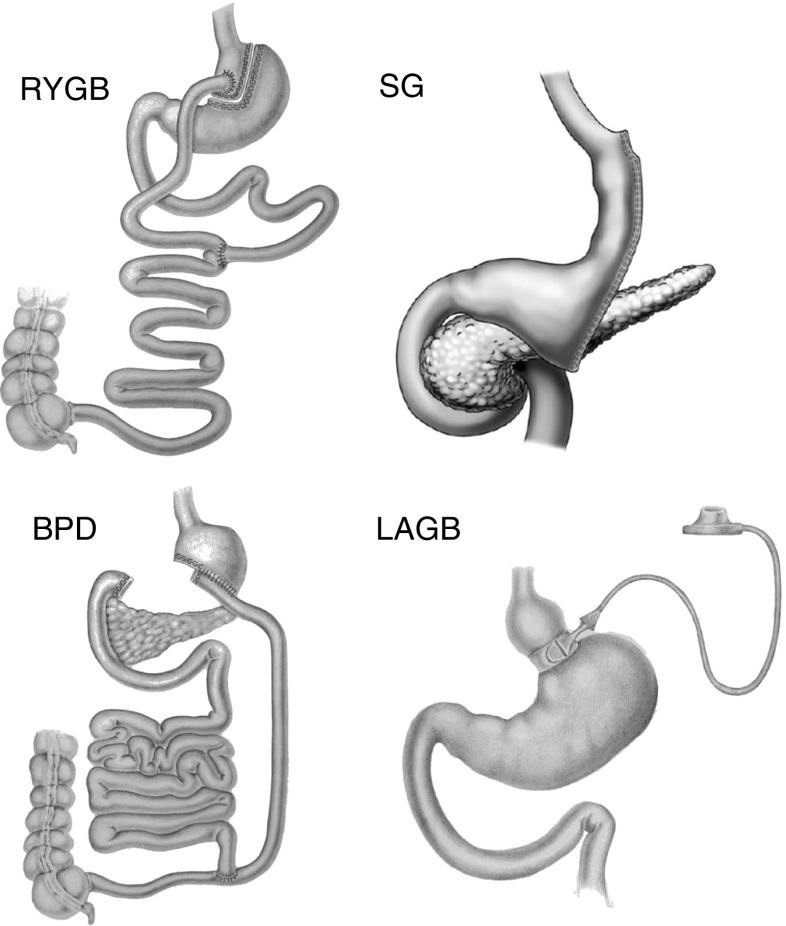
Glucose and lipids activate a neuronal axis connecting the intestine, the brain, and the liver through a *nutrient sensing*, probably located in the proximal jejunum, with subsequent inhibition of the hepatic glucose production. This nutrient sensing would be stimulated by undigested food delivered into the jejunum after RYGB, thus determining the reduction of the hepatic glucose production, a common feature of this operation. When the entire jejunum is bypassed, as it occurs after BPD, the secretion of putative insulin resistance factor/s might be inhibited with consequent normalization of insulin sensitivity

The three major bariatric surgical procedures, RYGB, BPD, and Sleeve Gastrectomy (SG), are described below. The major differences among these three types of operations are as follows. The stomach remnant is 30 ml in RYGB, 100 ml in SG and 400 ml in BPD. In the SG there is no intestinal bypass; in RYGB there is the exclusion of the duodenum and the first portion of the jejunum distal to the Treitz ligament from food transit; and in BPD, the duodenum, the whole jejunum and the first portion of the ileum are excluded from nutrient passage (see Fig. 1).

#### Roux-en-Y Gastric Bypass

This operation includes transection of the stomach with the linear staples just below the cardia in such a way as to create an upper pouch of about 25 ml based on the lesser curvature. The remaining stomach is left excluded from food transit. Next, the jejunum is divided at about 50 to 75 cm from the Treitz ligament: the distal end is connected to the small upper gastric pouch and the proximal end is joined to the jejunum some 70 to 150 cm distal to the gastric anastomosis, therefore fashioning a Roux-en-Y configuration.

Early mortality, 30 days after surgery, ranges between 0.3 and 0.5%. Excessive weight loss (EWL) is 60% at 3–5 years and 50% at 10 years [11].

#### Biliopancreatic Diversion

The operation entails distal gastrectomy, leaving a gastric pouch of about 400 ml, and closure of the duodenal stump. The ileum is then transected at 2.5 m from the ileocecal valve, and the distal end is brought up and anastomosed to the remaining stomach forming the so-called “alimentary tract”. The proximal end of the divided ileum, which carries the bile and the pancreatic juice, called “biliopancreatic tract”, is connected to the “alimentary tract” at 50 cm from the ileocecal valve in an end-to-side fashion. The last 50 cm of ileum, where food and biliopancreatic juice mix together, represents the “common tract”.

Early mortality is around 1%. Biliopancreatic diversion has repeatedly been shown to attain the best results in term of effectiveness and durability. EWL is 75% at 3 and 10 years [12].

#### Sleeve Gastrectomy

Sleeve gastrectomy is essentially the first step of BPD/duodenal switch where the stomach is resected along the greater curvature and fundus, leaving a small tube of stomach in continuity with the esophagus proximally and with the duodenum distally. The gastric volume is about 100 ml [13]. Early mortality is 0.19%. Mean EWL at 3 to 5 years is around 50% [13].

## Effect of Metabolic Surgery on Glucose Disposal and Insulin Secretion

Massive weight loss following RYGB largely ameliorates insulin sensitivity measured by the euglycemic hyperinsulinemic clamp (EHC) technique [14]. However, when whole body insulin sensitivity was studied by EHC within 1 month of RYGB, the results were controversial. While some authors did not find any significant improvement [14], other authors demonstrated a rapid amelioration of hepatic insulin resistance [15].

After BPD insulin sensitivity is normalized rapidly, within a matter of few days when the BMI is still unchanged. Initially, the effect of BPD on glucose disposal was attributed to the lipid malabsorption that accompanies this kind of surgery [16]. However, by shifting the temporal window earlier, just 1 and 4 weeks after BPD, when the BMI was not significantly changed, we observed that insulin sensitivity was already normalized, as shown by both the EHC and by an oral glucose tolerance test (OGTT) [16]. This effect, might be ascribed to the lack of gut mucosal stimulation by nutrients after the bypass of duodenal and jejunal tracts, which seem to secrete protein factors inducing insulin resistance.

Brozinick et al. [17] have reported that despite the marked in vivo insulin resistance observed for normal-glucose tolerant *db/db* mice during hyperinsulinemic clamps, their muscles were completely insulin responsive in vitro. Therefore, they suggested the presence of a humoral factor impairing the insulin action in vivo [17].

Indeed, proteins secreted by the duodenum and jejunum from both diabetic mice and insulin resistant humans induce insulin resistance in both normal mice and in muscle cells by stimulating the mTORC2 pathway [18•]. These gut conditioned medium proteins induce an over-basal phosphorylation of Akt on ^473^Ser, catalyzed by mTORC2, and a simultaneous reduction of its insulin-mediated phosphorylation on ^308^Thr, catalyzed by the Pyruvate Dehydrogenase Kinase 1 (PDK1), with impairment of Akt function in myocytes in vitro [18•]. Indeed, this same picture is a characteristic of the insulin resistant muscle and liver of rodents under a high fat diet [19].

Insulin secretion is increased after RYGB, but it is decreased after BPD as a consequence of the net improvement in insulin sensitivity [20•].

It is interesting to note that RYGB exerts its main effect in inducing diabetes remission through the reduction of hepatic glucose production [15] and increased insulin secretion, while BPD acts essentially by normalizing insulin sensitivity The first phase insulin secretion is promptly normalized after BPD [21••], whereas it is significantly improved, but does not return to normality, after RYGB [22].

The very-low calorie diet (VLCD) undergone in the weeks immediately following metabolic surgery might, indeed, contribute to remove the glucotoxicity and lipotoxicty present in diabetes. In fact, 2 weeks of VLCD alone improves the first phase of insulin secretion [23] and hepatic insulin sensitivity with reduction of glucose production [23]. However, we note that a VLCD does not improve peripheral insulin resistance [23]. These findings support the hypothesis that the reduction of gut mucosa stimulation by nutrients, as it happens after a VLCD, may not suppress the secretion of intestinal factor/s involved in insulin resistance, while the bypass of the duodenum and jejunum avoiding contact of nutrients with the gut mucosa does suppress this effect.

It is widely recognized that the primary defect in T2D is insulin resistance [24] with a relative deficiency in insulin secretion. This is the reason why some types of bariatric surgery, such as BPD, produce T2D remission by normalizing insulin sensitivity [7].

Contrary to individuals with type 1 diabetes, those with type 2 diabetes apparently do not show alteration in the number and dimension of pancreatic β-cells [25]. However, a more recent autopsy study reports that, compared with lean, non-diabetic individuals, T2D subjects have both a decrease of the relative β-cell volume/islet and a reduction of β-cell density [26]. Nevertheless, in spite of this reduced β-cell percentage in islets, the functional impairment of insulin secretion was shown to be mostly reversed by reducing islet oxidative stress [27]. Chronic supplementation of long-acting insulin in decompensated T2D subjects increases both first and second phase insulin secretion after an intravenous glucose tolerance test (IVGTT) [28]. After BPD, the first phase of insulin secretion, absent before the operation, is fully restored [21••]. A less impressive but still significant increase is also observed after RYGB [22], suggesting that β-cell dysfunction can drastically improve after bariatric surgery.

## Role of the Small Intestine in Glucose Homeostasis

Circulating glucose mainly derives from complex carbohydrates ingested with food. However, plasma glucose also originates from glycogenolysis or from gluconeogenesis from precursors such as lactate, pyruvate, amino acids, and glycerol.

Glucose concentration in the blood is regulated by a series of mechanisms involving the small intestine, which permit the circulating concentrations to be maintained within a narrow range. These mechanisms include gastric emptying, glucose absorption, and insulin secretion, all regulated by the small intestine. Next, we will discuss the effects of bariatric surgery on these glucose disposal steps.

## Gastric Emptying

Gastric emptying, accounting for about 35% of the variance of plasma glucose concentration at the peak after an OGTT [29], is regulated by the opening and closure of the pylorus that prevents the food mixed with gastric juices to enter the duodenum during mixing and crushing. In addition, a duodenal-gastric feedback mechanism—including vago-vagal reflex and hormonal signals, such as glucagon-like peptide-1 (GLP-1), peptide YY (PYY), and cholecystokinin (CCK) - intervenes in regulating gastric emptying. Gastric emptying influences the absorption and, thus, the circulating levels of glucose; in turn, blood glucose levels regulate the stomach’s emptying rate. In fact, glycaemia of 140 mg/dl or greater slows down the gastric emptying by 20 to 30% in both diabetic and healthy individual [30]. Hypoglycemia, in contrast, accelerates emptying, thus permitting more glucose absorption [31]. The accelerated transit of nutrients into the small intestine after bariatric surgery has been regarded by some authors as a major cause of diabetes remission, since it stimulates GLP-1 and insulin secretion [32].

Subtotal gastrectomy and gastric exclusion, as after SG and RYGB, are often accompanied by a dumping syndrome and late hypoglycemic episodes. In the dumping syndrome, neuro-vascular and gastrointestinal symptoms, related to the rapid gastric empting and increased intestinal motility, emerge within 30 min from the meal, while late symptoms are related to hypoglycemia (late dumping) and appear 2 h or more after the meal. Bender et al. [33] noted that the dumping syndrome and, in particular, hypoglycemia occur also in patients with intact stomachs and feeding jejunostomies, suggesting that it is driven by the bypass of the duodenum and the first portion of the jejunum, or by the direct delivery of nutrients into the distal jejunum.

The most accepted explanation for dumping hypoglycemia is the excessive insulin secretion secondary to a rapid absorption of simple carbohydrates with a large early peak of blood glucose. However, many studies demonstrated that when hyperglycemia was induced in subjects with partial gastrectomy by intravenous glucose infusion in order to mimic the OGTT curves, no reactive hypoglycemia was elicited. In fact, i.v. infusion of glucose does not stimulate insulin secretion in comparable amounts, nor is it followed by hypoglycemia [34]. It is possible that oral glucose overstimulates insulin secretion by inducing GLP-1 secretion. Indeed, the simultaneous infusion of glucose and GLP-1 in healthy volunteers, mimicking the glycaemic and insulin peaks in patients with dumping syndrome, does provoke hypoglycemia [35].

Reactive hyperinsulinemic hypoglycemia with neuroglycopenia following gastric bypass has been described in less than 100 cases and it is related to nesidioblastosis, characterized by diffuse hyperplasia and hypertrophy of pancreatic β-cells [36]. However, what is unclear is whether congenital or undiagnosed nesidioblastosis was already present before bariatric surgery or if it developed as a consequence of the operation. If nesidioblastosis preceded these gastric bypass operations, it is possible that the progressive weight gain leading to these surgeries may have resulted from the life-long intake of multiple daytime meals rich in carbohydrates prompted by patients’ need to prevent hypoglycemic episodes.

## Glucose Absorption and Nutrient Sensing

The enterocytes, representing the majority of the intestinal epithelial cells, are highly differentiated cells with specific polarization. Through the apical brush-border membrane they transport nutrients towards the baso-lateral membrane domain and successively to the intestinal capillary system.

Enterocyte transport of glucose and galactose is performed via the sodium/glucose co-transporter 1 (SGLT1). The expression of SGLT1 increases from the duodenum down to the ileum and it is up-regulated by the endoluminal concentration of glucose suggesting its function as the *intestinal glucose sensor*. Parker et al. [37] have proposed that glucose uptake by SGLT1 can stimulate the release of GLP-1 by L-cells and GIP by K-cells given that the use of phlorizin, a competitive blocker of sodium-glucose transporters, suppresses incretin secretion.

Once in the enterocytes, glucose is moved to the interstitial space by the basolateral glucose transporter (GLUT) 2, a bidirectional transporter moving glucose outside or inside the intestinal epithelial cells depending on the glucose gradient.

The jejunal infusion of glucose in normal rats reduces hepatic glucose production, an effect that is reversed by phlorizin blockade of sodium-glucose transporters located in the mucosa of the small intestine [38]. This jejunal nutrient sensing is also required for the rapid resolution of diabetes in streptozotocin treated rats after duodenal-jejunal bypass [39]. During refeeding, the jejunal nutrient sensing is disrupted, resulting in increased circulating glucose levels.

Nutrient sensing and glucose homeostasis seem to be regulated by the ventromedial hypothalamus (VMH), where insulin receptors are expressed [40]. VMH insulin receptor knockdown (IRkd) mice develop hepatic insulin resistance, glucose intolerance, increased glucagon, and impaired insulin secretion [40]. RYGB reduces hepatic glucose production by 58% in high fat diet rats independently of body weight reduction, but IRkd prevents this improvement suggesting that an increased VMH sensitivity to insulin could be mediating this effect of bariatric surgery; however, the improvement of peripheral insulin sensitivity was unaffected by central insulin receptor knockdown [40]. These data were confirmed in Zucker insulin resistant rats [41] where rapid normalization in hepatic gluconeogenic capacity and basal hepatic glucose production required intact vagal innervations, while this was unnecessary for restoration of insulin sensitivity.

Interestingly, bypass of the duodenum and the proximal jejunum, as obtained by infusing simple nutrients into the mid jejunum, is associated with a significant improvement of whole body insulin sensitivity in both diabetic and non-diabetic individuals [42].

Duodenal-jejunal bypass in congenitally diabetic rats significantly improves glycemic control [43]. Avoiding contact of nutrients with the duodenal-jejunal mucosa and their absorption, the endoluminal sleeve also improves glucose control and tolerance in humans.

## Incretins

The enteroendocrine cells, although in a much lower number than enterocytes, represent the largest endocrine organ in the body. Until now, at least twenty different subpopulations of enteroendocrine cells have been identified. Amongst them, K-cell density is maximal in the duodenum, progressively declining through the jejunum and ileum. The opposite is observed with L cells. While K cells secrete glucose-dependent insulinotrophic peptide (GIP), L cells produce GLP-1 and GLP-2, although GLP-1 and GIP are co-localized in a subset of intestinal endocrine cells in humans and pigs [44].

Nutrient-driven stimulation of GIP release in the duodenum enhances the release of GLP-1 distally in the ileum. In addition to GIP, many other neuropeptides and neurotransmitters increase the release of GLP-1 from L cells, namely gastrin-releasing peptide, calcitonin gene-related peptide, and acetylcholine, the latter acting via muscarinic receptors.

Prohormone convertase 1 produces glicentin, oxyntomodulin, GLP-1, and GLP-2 from proglucagon in L cells. GLP-1 stimulates insulin secretion, accounting for ca. 70% of the insulin secretion after oral glucose in the presence of high levels of glucose. The hormone binds the GLP-1 receptor, which is a member of the G protein–coupled receptor family, inducing the production of cyclic AMP. Cyclic AMP stimulates protein kinase A (PKA), involved in the activation of the insulin gene transcription by glucose, and activates the guanine nucleotide exchange factor II (GEFII or Epac2) raising intracellular Ca^++^ concentration, fostering the release of insulin stored vesicles. GLP-1 receptor knockout mice (*GLP-1r−/−*) show glucose intolerance and reduced insulin secretion after a glucose load [45].

In patients with T2D, GLP-1 improves both early and late phases of the insulin response to glucose and suppresses glucagon secretion by the pancreatic α-cells, leading to reduced endogenous glucose production from the liver.

Similarly to GLP-1, GIP enhances intracellular cyclic AMP generation and inhibits ATP-sensitive K^+^ channels, thereby increasing intracellular Ca^++^ levels with consequent stimulation of insulin secretion. GIP has a role in adipocyte metabolism, since *Gipr* ^*−/−*^ mice show a reduction of fat depots and are less prone to increase weight under a hypercaloric diet [45]. In addition, GIP deficient *ob/ob* mice are leaner than their wild type littermates and show an improved glucose tolerance. The GIP antagonist Pro [3] GIP induces weight loss in diabetic mice and improves glucose tolerance [46].

Duodenal-jejunal bypass (DJB) in congenitally diabetic Goto-Kakizaki rats determines a significant increase in the pancreatic islet β-cell area and a decrease of islet fibrosis [47]. These morphologic features are associated with a functional increase of insulin secretion. GLP-1 rises in the plasma of diabetic rodents after DJB, derived from an increased population of cells co-expressing GIP and GLP-1 in the jejunum anastomosed to the stomach. Therefore, the bypass of the duodenum and jejunum by food transit enhances the differentiation of intestinal stem cells into intestinal enteroendocrine cells producing GLP-1, with subsequently reduced β-cell deterioration.

A large increase of incretin secretion was observed in diabetic, obese subjects following RYGB. Their blunted effect was normalized just 1 month after the operation. This outcome was not attributable to the low calorie intake following metabolic surgery; in fact, incretin secretion after an OGTT was unaffected by a low calorie diet matching the caloric intake in RYGB patients [48].

Contrary to what happens after RYGB, BPD does not overstimulate incretin secretion [22] and it is characterized by reduced insulin secretion to match the normalization of insulin sensitivity.

In a recent study, the effect of SG was investigated in GLP-1 receptor knockout mice, showing that the absence of GLP-1 receptor and, thus, of GLP-1 action, is not required for obtaining the improvement of the glucose disposal, which was similar to that observed after bariatric surgery in wild type rodents. These data suggest that the effect of bariatric surgery on glucose metabolism cannot be mediated by the increased GLP-1 secretion alone.

## Ghrelin

Ghrelin is a polypeptide composed of 28 amino-acid residues principally secreted by the X/A-like cells within the gastric oxyntic glands. Ghrelin secretion is increased in the fasting state and suppressed by feeding, and exerts an orexigenic action. Obese subjects show a reduction of ghrelin secretion after meals [49]. Diet-induced weight loss is associated with a marked increase in the circulating levels of ghrelin after the meals, while a striking suppression of its secretion is observed after RYGB and SG [50]. Its effect in increasing appetite is mediated via the stimulation of NPY/agouti-related peptide (AgRP) co-expressing neurons within the arcuate nucleus of the hypothalamus. SG delayed T2D onset in the University of California Davis-T2D rat, independently of body weight loss. This effect was mediated either by decreased circulating ghrelin concentrations or increased circulating levels of bile acids, adiponectin, and GLP-1 [51]. Other studies highlight the role of decreased ghrelin secretion as a possible mechanism of action of metabolic surgery [50].

However, vertical SG is effective in improving glucose tolerance in both wild-type and ghrelin knockout mice when exposed to a high-fat diet for 10 weeks before surgery [52]. This shows that, at least in rodents, ghrelin does not play a key role in the remission of diabetes that follows bariatric surgery, but it is rather an epiphenomenon related to the partial gastrectomy.

## Gut microbiota and glucose metabolism

The human gut microbiome is composed of more than 10^12^ cells per gram of feces, the majority of which are prokariotes. Its biodiversity is enormous as it is under the control of approximately 3 million genes.

The Human Microbiome Project Consortium has studied 242 healthy subjects, 129 men and 113 women. Among a series of other samples, stool specimens represented the microbiota of the lower gastrointestinal tract. The majority, 90% of the mammalian gut microbiota, belongs to two phyla, the Bacteroidetes and the Firmicutes. Lactobacillus and Streptococcus, which are acid-resistant, are the only two microorganisms that can survive in the stomach. The number of bacteria increases distally throughout the small intestine so that in the ileum and, in particular, in the colon it reaches a peak.

Microbiota exert a series of actions in the intestinal medium from bile acid metabolism to the regulation of intestinal permeability and the modulation of inflammation [53]. In the large intestine, anaerobic bacteria deconjugate bile acids to form secondary bile acids. Primary bile acids bind the nuclear farnesoid-X receptor (FXR) while the secondary bile acids, deoxycholic and lithocholic acids, bind the G protein-coupled receptor (GPCR) TGR5. Interestingly, FXR impairs, whereas TGR5 promotes, glucose homeostasis. Therefore, a lack of transformation of primary into secondary bile acids negatively affects glucose metabolism. In addition, the activation of TGR5 in adipose brown tissue increases energy expenditure and protects against diet-induced obesity [53].

The use of bile acid sequestrants in diabetic patients improved glycemic control [54], possibly through the enhancement of GLP-1 secretion as shown in experimental animals.

As stated above, primary bile acids bind the nuclear receptor FXR that regulates the expression of genes involved in lipid and carbohydrate metabolism and in energy expenditure. The activation of FXR inhibits the sterol regulatory element binding protein 1-c (SREBP1c) that mediates hepatic lipogenesis and activates the synthesis of apolipoprotein CII (apo CII), resulting in the subsequent increase of triglyceride clearance from the circulatory stream. In addition, FXR inhibits hepatic apo CIII production with a consequent increase of lipoprotein lipase activity [53]. The activation of FXR by the synthetic agonist GW4064 or overexpression of hepatic FXR by adenovirus-mediated gene transfer in the liver markedly reduces blood glucose levels in both *db/db* and wild-type mice [55]. FXR deficiency in genetically obese (*ob/ob*) mice and in diet-induced obese mice is associated with a reduction of adipose tissue, increased insulin sensitivity and increased glucose disposal [56].

The first evidence of the role played by the intestinal microbiota in energy balance was provided by Backhed et al. [57], whose seminal paper demonstrated that germ-free animals, which were slimmer than normal littermates, became more obese once they received coecal bacteria of the latter despite no change in food consumption. This effect was secondary to more efficient food energy utilization by the bacteria that colonized the intestine of germ-free mice. An inverse relationship of Firmicutes to Bacterioidites in lean individuals, with increase of the former and reduction of the latter, represents a typical feature of obesity in both animals and humans.

Relatively few data are available in the literature regarding the stool microbiota composition after bariatric surgery. Zhang et al. [58] found a net increase of Gammaproteobacteria and a reduction of Firmicutes in subjects who had undergone RYGB as compared with morbidly obese ones who did not receive the operation; however, the number of participants was very limited at only three. As an additional limitation, these individuals were not studied preoperatively.

In Wistar rats operated with RYGB, Li et al. [59] showed a significant increase of Proteobacteria, and in particular of Enterobacter hormaechei, and a reduction of Firmicutes and Bacteroidetes, in comparison to sham-operated animals. Gut microbiota composition can determine the efficacy of energy harvest from food. In fact, a 6-week, energy-restricted, high-protein diet, followed by a 6-week weight-maintenance diet, reduced adipose tissue and systemic inflammation in overweight or obese individuals by correcting a putative loss of richness in low gene-count individuals [60].

Although it is possible that gut microbiota changes after bariatric surgery play a role in the improvement of glycemic control and amelioration of the insulin resistance status, further research is necessary to determine the degree to which such changes account for the metabolic benefits of bariatric surgery.

## Conclusions

The small intestine exerts a primary role in glucose homeostasis, as the jejunum senses the nutrients and regulates hepatic glucose production and the entire small gut secretes both GLP-1 and GIP, thereby enhancing insulin secretion. These physiological actions are magnified by the intestinal manipulations performed during bariatric surgery, with subsequent increase of insulin sensitivity, as happens after BPD, or an increase in insulin secretion as a consequence of incretin hypersecretion, as it occurs after RYGB. The possible mechanism of action of bariatric surgery on diabetes is summarized in Fig. 1. We hypothesize that bypass of the duodenum increases the stimulation of a jejunal nutrient sensor that is transmitted to the hypothalamus, having a negative feedback effect to reduce liver glucose output and improve hepatic insulin resistance. Furthermore, the exclusion of duodenum and the entire jejunum from food transit suppresses the secretion of intestinal hormones responsible for inducing peripheral insulin resistance, with a consequent improvement of glycemic control in T2D individuals. In addition, the changes of gut microbiota after bariatric surgery may contribute to the amelioration of hepatic insulin sensitivity.
